# Some of the Hardships of the General Practitioner of Medicine, and a Suggestion for Relief

**Published:** 1894-02

**Authors:** R. P. Talley

**Affiliations:** Temple, retiring President; Temple, Tex.


For Texas Medical Journal.

### SOME OF THE HARDSHIPS OF THE GENERAL PRACTITIONER OF MEDICINE, AND A FEW SUGGESTIONS FOR RELIEF.

President's Address to Austin District Medical Society, Dec. 21, 1893. R. P. Talley, M. D., Temple, retiring President. Referred to Publishing Committee and ordered published.

GENTLEMEN:—As retiring President of your Society, it devolves upon me, in accordance with custom, to deliver an address; and were I to strictly follow custom, I should select for my subject "Our Noble Profession," or "The Grand Achievements of Medical Science," or "The Grandeur and Glory of Being a Doctor," or something on that order; but so much has been said on all those subjects, that there remains nothing for me to add; they have been pretty well used up. I have, therefore, struck out on a new line, and with your patient indulgence, I will try to lead you through certain new paths—heretofore untrodden, along which grow not so many or so bright flowers; not so inviting; and I may tell you some things that may not be so pleasant. First. I have something to say about the specialists in medicine and in surgery; and will enumerate here only a few:

1. The eye, ear, nose and throat *specialist.*
2. The gynecologist *specialist.*
3. The railway surgeon *specialist.*
4. Whisky, opium, etc., *specialist.*
5. Dentist, or oral surgeon *specialist.*
6. Orificial surgeon *specialist.*
7. The Arkansas Hot Spring *specialist.*
8. The self-constituted midwives, and on down the line to the street medicine man, the Christian science doctors, the pile doctors, the Keeley's, etc., about whom and which I shall have nothing to say on this occasion.

Now, perhaps any one belonging to any of these classes would, at first thought, earnestly and honestly object to being considered a hardship on the work of the general practitioner of medicine. But I want to see the general practitioner of much experience who will not agree substantially with me. Let me here insist that I am a full believer in the importance of good specialists in all the subdivisions of the whole range of scientific medicine. But I want the specialist in the specialist's place only. It is a serious hardship on the family physician to have, for instance, the oculist to treat any eye trouble, from a singed eye lash up to the most difficult operations known to the science of surgery, or the gynecologist to stitch up insignificant lacerations of the cervix uteri, or perineum, at from one to one hundred dollars a stitch—lacerations that the family physician regarded properly as ten times less trouble and danger to life and health than the surgical treatment adopted by the specialist. Of course, all calling themselves specialists are not guilty to such an extent; but it is a well know fact that too many are, and of even greater. It is hard enough on the general practitioner when the specialist does only such practice as properly requires the skill of a specialist; but when they set up as specialists, and then try to ride this "ad." into the practice of the whole science of medicine as some of them do, it is, in my judgment, not quite fair. They get an "ad." on the word "specialist," and then comes the reputation—the notoriety—as a specialist; and then comes the practice too, it may be, a magnificent display office, where they are at ease and comfortable, with regular meals, plenty of sleep, snugly protected from foul weather, and the fearful dangers of all sorts of contagious diseases common to general practitioners. So we see this reputation on the word specialist is something not to be dispised by every one. But for me, let me parody Madame Roland by exclaiming "Reputation, Oh! reputation, how many crimes are committed in thy name."

Some of the most noted specialists are so anxious to do poor humanity good (charity of course beginning at home), that they are willing to, and even advocate taking into the medical fold all the medical isms and pathies of the age, ostensibly for humanity's sake, but in fact, to get more grinding for the specialty mill. In a great measure, it is the office and duty of the family physician to prescribe or proscribe the specialist just as he would a dose of calomel, or a visit across the sea. In fact, it is my opinion that the family physician does not advise his patients, as a rule, as much as he should, as to the proper beginning and ending of the functions of the ordinary specialist. It becomes his moral, as well as his professional duty, to advise his patients not to go to a specialist with every frivolous trouble, and thereby save time, money, trouble, etc. I want it well understood that I am not criticising the specialists of soul and brain. The specialist proper is a general practitioner *plus* the capacity as a specialist, and knows where the functions of his work as such, begin and end. On one occasion while Prof. Roosa, of New York, was lecturing on diseases of the eye, "a case" was sent in by his assistant as clinical material. The professor gave an examination and announced to the class that the case was not one of diseased eyes, but one of diseased kidneys—a case of Bright's disease. The next lecture was by Prof. Loomis, on diseases of the heart. His assistant sent in as clinical material the same case. Prof. Loomis examined the case carefully before the same class Prof. Roosa had just lectured to, and made the very same diagnosis without knowing anything of what had just been decided by the preceding lecturer. He was even so astonished when the class of about six hundred students set up a deafening cheer that he stopped his lecture and made inquiry, as he said, "what have I said that is so funny?" Of course this accident, as it were, gave and thoroughly impressed an important lesson. But these men were not embyro specialists, or specialists from a commercial standpoint, but specialists for the good of science.

What has been said in regard to the eye, ear, nose and throat specialist will apply substantially to the majority of other specialists, except perhaps the railway surgeon specialist. This is comparatively a new specialty, but is growing in all directions remarkably rapidly. This class now has a National, several State and local organizations, from which all doctors are excluded who do not hold an appointment, either directly or indirectly by some railway corporation. These organizations are perhaps all right morally, financially, and according to the laws of the land, but nevertheless, a hardship on the general practitioner. I will here mention one as an illustration—one with which I am somewhat painfully acquainted. But let it be known that it is not the organization, abstractly speaking, to which I make exceptions, but the *manner* of the backing it gets. I refer to "The ——— ———————, which has just lately been organized out of the physicians holding official positions with the Gulf, Colorado & Santa Fe Railway Company. This company has what is known as the Gulf, Colorado & Santa Fe Railway Company Hospital Association, which is supported by a monthly tax upon all the employes of the company, and is ostensibly controlled by a board of five trustees, but in fact, is under the absolute power of the company, indirectly through its superintendent. This Hospital Association is a chartered institution, but chartered in such terms and way as to require that the superintendent, attorney and surgeon of the company shall be members of the board of trustees; the members have some say as to who shall be the other two members of the board, thus the company makes the majority of the board of trustees, and hence controls in fact, the Hospital Association, notwithstanding any objections the members—those are taxed to support it—may at any time urge. So we see that the essential and important office ot the chief surgeon of this Hospital Association is the office as chief surgeon of the railway company. Therefore it is easy to see that the chief surgeon of the Hospital Association is, in fact,

*of, and for the railway company*, and indirectly for those who are "taxed without representation" to support as may seem the interest of the company. We see the advantage ground the chief surgeon of *these* institutions has, backed as he is, and created by a powerful financial corporation which is, in fact, his patient for first consideration. I may mention here, that these chief surgeons have their "subs" "all along the line," and out of these comes the material for these organizations of railroad doctors.

Now let us grant that thus far we have not made any serious charges against the chief surgeons and their satelites as affecting materially the welfare of the general practitioner of medicine. And we would have no grounds for doing so, and would be the last to even try to, if these fellows would stick strictly to their specialty—practice for the railroad companies and their employes. But such is not the case; especially with the chief surgeons and some of their subs. For instance, the surgeon in chief of the G. C. & S. Ry. Co., who is also chief surgeon of the G. C. & S. F. Ry. Co. Hospital Association, has his assistant of his own creation, both of whom get a handsome salary of—it is hard to find out just how much—to do the practice for the hospital of this association and the employes living in the same town, who get sick or otherwise disabled strictly in the line of, and on duty as such. Thus it is seen that these surgeons are not required to do any and all practice for the employes and look to the association for the pay. Now here is the "rub:"

1. These surgeons are backed by a powerful financial corporation.

2. They get a handsome salary for a fraction of their professional time.

3. They are not required to give their whole time and energies to their specialty.

4. Out of all they get a big "ad." by which they step from the practice from the employes to their families and friends, as well as to the practice for the employer not allowed under the rules of the Hospital Association.

This is all very nice and desirable for the railroad doctors, but I think it is a devil of a hardship on the "other fellows."

I would say something about the Arkansas Hot Springs specialist, but we all already know him too well. His virtues as such are advertised on every telegraph pole, hog pen, etc., throughout the entire country. Unless we can make people understand that water can be made as hot in Texas as it can in

Arkansas, our patients will continue to slip out and pay ten times as much for their troubles as if they would try in any sort of good faith their family physicians.

Now, I have rambled enough about the specialist; and in doing so I mean no harm or disrespect to any one or class, but simply to mention what I think are causes of trouble, that we may thereby perhaps do better.

Now, I want to say something about what I conceive to be the greatest of all the hardships with which the practitioner of medicine has to contend, to-wit: The excess in the number of legally constituted doctors of medicine, and their general deficiency in qualification.

In regard to number, I will mention my own locality, taking it for granted that doctors, as to quantity, are like water, i. e., seek their own level. In my town, of something less than six thousand inhabitants, we have twenty-two doctors, and in the country around, there are twenty-four more within ten miles. Some authority has estimated that in the army, in time of war, one physician can serve five hundred soldiers, and that in civil life one thousand and five hundred patrons are required to support one general practitioner. According to this estimate, in my town we have eighteen out of twenty-two doctors to spare, and if we cannot get rid of this excess, somebody is hurt. This excess in number leads to all manner of "sharp work" to get practice. Such a crowded population of doctors reminds me of a crowded population of poor people, where we find the hot-beds of all sorts of pernicious diseases, moral as well as physical. Why, in too many localities our doctors are crowded, as well as illy bred as doctors, until the slime of local rivalry rises mountain high. If some local brother steps a little aside from what some other local brother thinks is ethical, he forthwith gets all sort of infamy heaped upon his professional head. Who ever heard of two or three doctors getting together, singing a song and praying for some professional sinner, who had perhaps even sinned without knowing it. There is only one occasion when doctors may get together and sing and pray for one another, to-wit: When one dies, they can sometimes meet around his grave, and say amen, and then pass resolutions of condolence with a prayerful and perfect unanimity.

The proverb that "good articles are always put up in small quantities," seems to apply to doctors of medicine as well as to anything else. If good doctors in this country are not put up in small quantities, I fail to understand properly the subject. And what is still worse, they are put up in such small quantities that our mongrel, miserable, mixed population fails to recognize or care for the difference in the good or the worthless. In this country a doctor is a doctor, and equal to any other doctor, especially if a specialist,—no difference as to ism or pathy or education. Strange it is that our so-called best people will patronize the miserable, low-down, itinerating street quack doctor, even sometimes clandestinely through an ignorant servant, or otherwise, if pressed for a chance, instead of trusting to any one who has spent a lifetime to try to know something in fact about the science of medicine. I could mention two senators now in the Congress of the United States who have sent, or allowed members of their families to go across the country (the United States) to consult a specialist in the Christian faith for their real, or it may be, their supposed fleshly ills. This condition of things is well calculated to make a man sick, who has spent his whole life in the study of medicine. If you please, where is the foundation upon which to build an energy and ambition to become a physician in the true meaning of the term?

For many years I have not advised a young man to undertake the study of medicine as a profession unless I thought he had three special qualifications, to-wit: (1) a good moral constitution, (2) a good brain, and (3) prospectively in such financial circumstances as to enable him to live independent of any income from his professional work. One of the best physicians I have ever known owned several thousand dollars worth of laboratory outfit, etc., for the study of medicine, and was at the same time independent and indifferent as to any money income from his professional services. That plenty of financial income for our professional services is an all important motor power, there is no sort of doubt; but if we have to have it, we are too often painfully left, and hence physicians, like other men, when about to drown "catch at straws," and do many other little things, which I believe belong, as a rule, under the wings of the angels of charity, instead of being spit out in a spiteful manner by those who glory in the spirit of who can *least* work and *least* agree.

Now, I am not ambitious to become a faith-breaker in changing the rule of these presidential addresses from all glory! glory! and hallelujah! by taking part at the dark side of the physician's life. I fully believe that I am substantially correct in what I have said on this subject. As to the causes of all this trouble,

I have not the time, and am not prepared to enter into much discussion or investigation. It seems to me that in enumerating the facts, the causes are indicated all along the line. To be charitable and just, I cannot believe that premeditated meanness and viciousness in human nature have had, comparatively, much to do in causing the common hardships of the general practitioner of medicine. It seems to me that the words, *want of education*, will cover all the causes, and the words *higher education* suggest the only relief to be hoped for. Higher medical education has always been warmly advocated by everybody having any knowledge of the subject. This has done great good, no doubt. By higher and still higher medical education, the medical mills cannot grind just any ignoramus into a full-grown, legally-authorized doctor of medicine; thereby decreasing the quantity and improving the quality of the profession of medicine, which is all well enough so far as it goes. But this is not all the education needed to help the doctors. The people, especially in our form of political government, must also have a higher education,—an education by which they may know and also appreciate an educated physician.

Gentlemen, for your kindness and patience to hear this, my dark-sided picture of professional life, and for allowing me to serve you as your President, I thank you.

